# Crystal structure, Hirshfeld surface analysis and inter­action energy calculation of 1-decyl-2,3-di­hydro-1*H*-benzimidazol-2-one

**DOI:** 10.1107/S2056989021004291

**Published:** 2021-04-27

**Authors:** Younesse Ait Elmachkouri, Asmaa Saber, Ezaddine Irrou, Bushra Amer, Joel T. Mague, Tuncer Hökelek, Mohamed Labd Taha, Nada Kheira Sebbar, El Mokhtar Essassi

**Affiliations:** a Laboratoire de Chimie Appliquée et Environnement, Equipe de Chimie Bioorganique Appliquée, Faculté des Sciences, Université Ibn Zohr, Agadir, Morocco; b Laboratoire de Chimie Organique Hétérocyclique URAC 21, Pôle de Compétence Pharmacochimie, Av. Ibn Battouta, BP 1014, Faculté des Sciences, Université Mohammed V, Rabat, Morocco; c Faculty of Medicine and Health Sciences, Sana’a University, San’a, Yemen; d Department of Chemistry, Tulane University, New Orleans, LA 70118, USA; e Department of Physics, Hacettepe University, 06800 Beytepe, Ankara, Turkey

**Keywords:** crystal structure, C—H⋯π(ring) inter­action, di­hydro­imidazole, Hirshfeld surface analysis

## Abstract

The title mol­ecule, C_17_H_26_N_2_O, adopts an L-shaped conformation, with the straight *n*-decyl chain positioned nearly perpendicular to the di­hydro­benzimidazole moiety. The di­hydro­benzimidazole portion is not quite planar as there is a dihedral angle of 1.20 (6)° between the constituent planes. In the crystal, N—H⋯O hydrogen bonds form inversion dimers, which are connected into the three-dimensional structure by C—H⋯O hydrogen bonds and C—H⋯π(ring) inter­actions. Hirshfeld surface analysis indicates that the most important contributions for the crystal packing are from H⋯H (75.9%), H⋯C/C⋯H (12.5%) and H⋯O/O⋯H (7.0%) inter­actions. Based on computational chemistry using the CE–B3LYP/6–31 G(d,p) energy model, C—H⋯O hydrogen bond energies are −74.9 (for N—H⋯O) and −42.7 (for C—H⋯O) kJ mol^−1^.

## Abstract 2

The title mol­ecule adopts an L-shaped conformation with a straight alkyl group. In the crystal, N—H⋯O hydrogen bonds form inversion dimers, which are connected into chains extending along the *b*-axis direction.

## Chemical context   

Benzimidazol-2-one derivatives constitute an important class of heterocyclic systems. They are used as precursors for the preparation of novel N-substituted benzimidazol-2-one deriv­atives with potential biological and pharmacological properties (Lakhrissi *et al.*, 2008 ▸; Saber *et al.*, 2019 ▸; Mamedov *et al.*, 2017 ▸), including anti­tumor (Khodarahmi *et al.*, 2005 ▸), anti­bacterial (Saber *et al.*, 2020*a* ▸; Vira *et al.*, 2010 ▸), anti-HIV (Barreca *et al.*, 2007 ▸), and anti­trichinellosis (Mavrova *et al.*, 2005 ▸) activities.
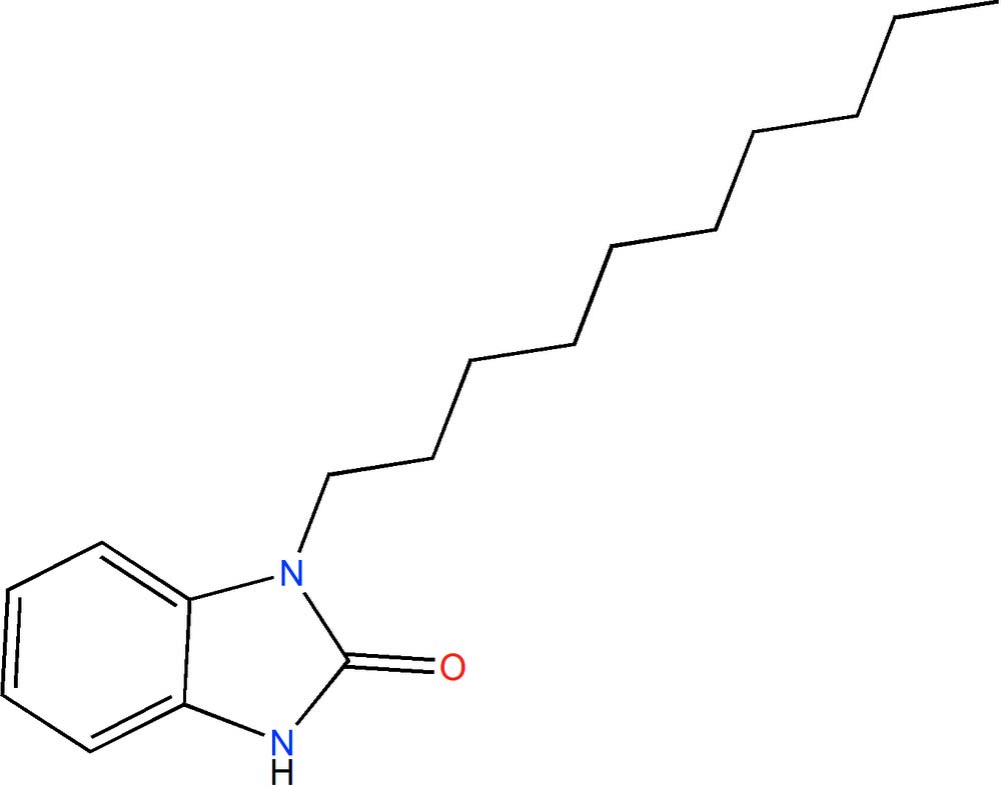


In continuation of our investigations on the synthesis, physico-chemical characterization and biological properties of novel N-substituted benzimidazol-2-one derivatives, we have studied the reaction of 1-bromo­decane with 1-isopropenyl-1*H*-1,3-benzimidazol-2(3*H*)-one under phase-transfer catalysis conditions (Saber *et al.*, 2020*b* ▸; Srhir *et al.*, 2020 ▸), We report herein the synthesis, and the mol­ecular and crystal structures along with the Hirshfeld surface analysis and the inter­molecular inter­action energies of the title compound, C_17_H_26_N_2_O, (I).

## Structural commentary   

**Figure 1:**
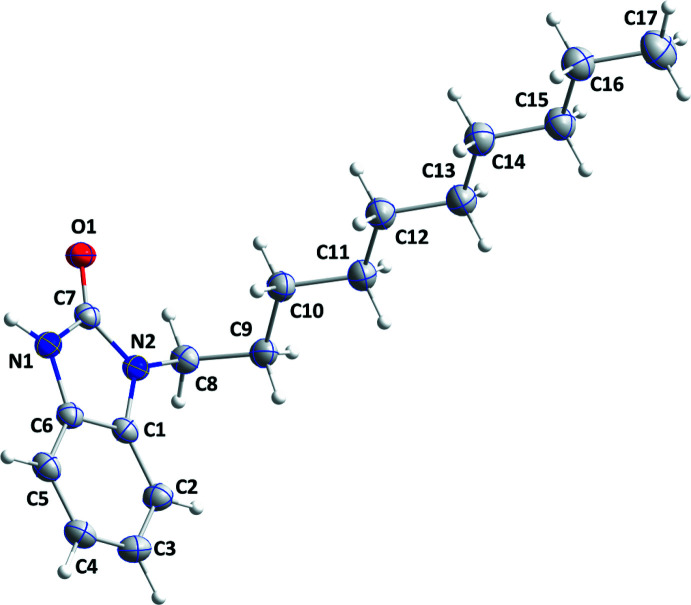
The asymmetric unit of the title compound with the atom-numbering scheme. Displacement ellipsoids are drawn at the 50% probability level.

The title mol­ecule adopts an L-shaped conformation with the straight *n*-decyl chain arranged nearly perpendicular to the di­hydro­benzimidazole portion, as indicated by the C1—N2—C8—C9 torsion angle of −75.91 (12)° (Fig. 1 ▸). The di­hydro­benzimidazole portion is not planar, as indicated by the dihedral angle of 1.20 (6)° between the constituent planes.

## Supra­molecular features   

**Figure 2:**
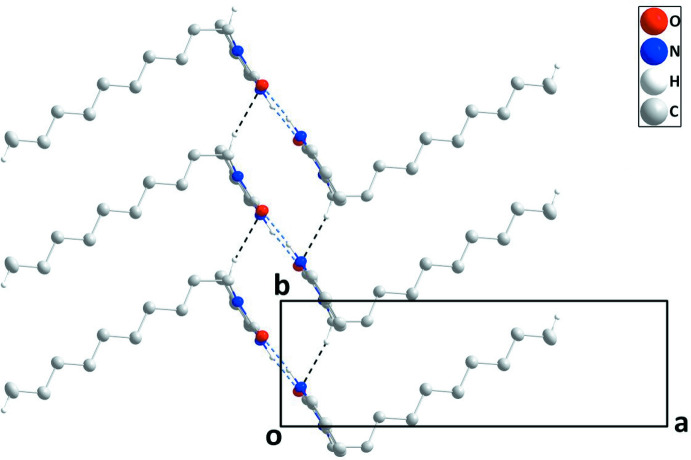
A portion of one chain viewed along the *c-*axis direction with N—H⋯O and C—H⋯O hydrogen bonds depicted, respectively, by blue and black dashed lines. H atoms not involved in hydrogen bonding were omitted for clarity.

**Figure 3:**
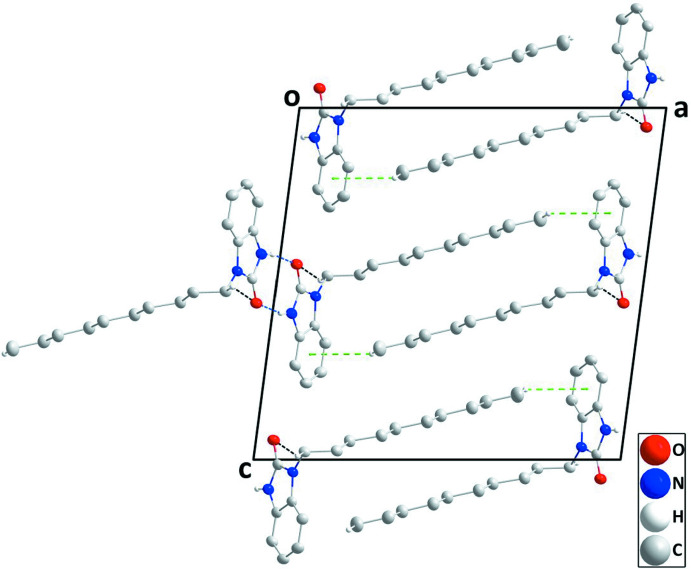
Packing viewed along the *b-*axis direction with hydrogen bonds depicted as in Fig. 2 ▸ and C—H⋯π(ring) inter­actions by green dashed lines. H atoms not involved in hydrogen bonding were omitted for clarity.

In the crystal of (I), inversion dimers are formed by N1—H1⋯O1 hydrogen bonds (Table 1 ▸) that are linked into chains extending parallel to the *b* axis by C8—H8*A*⋯O1 hydrogen bonds (Table 1 ▸, Fig. 2 ▸). The alkyl groups extend from both sides of the chain and inter­calate with alkyl groups of adjacent chains while linking them together through C17—H17*C*⋯*Cg*2 inter­actions (Table 2 ▸, Fig. 3 ▸).

## Hirshfeld surface analysis   

**Figure 4:**
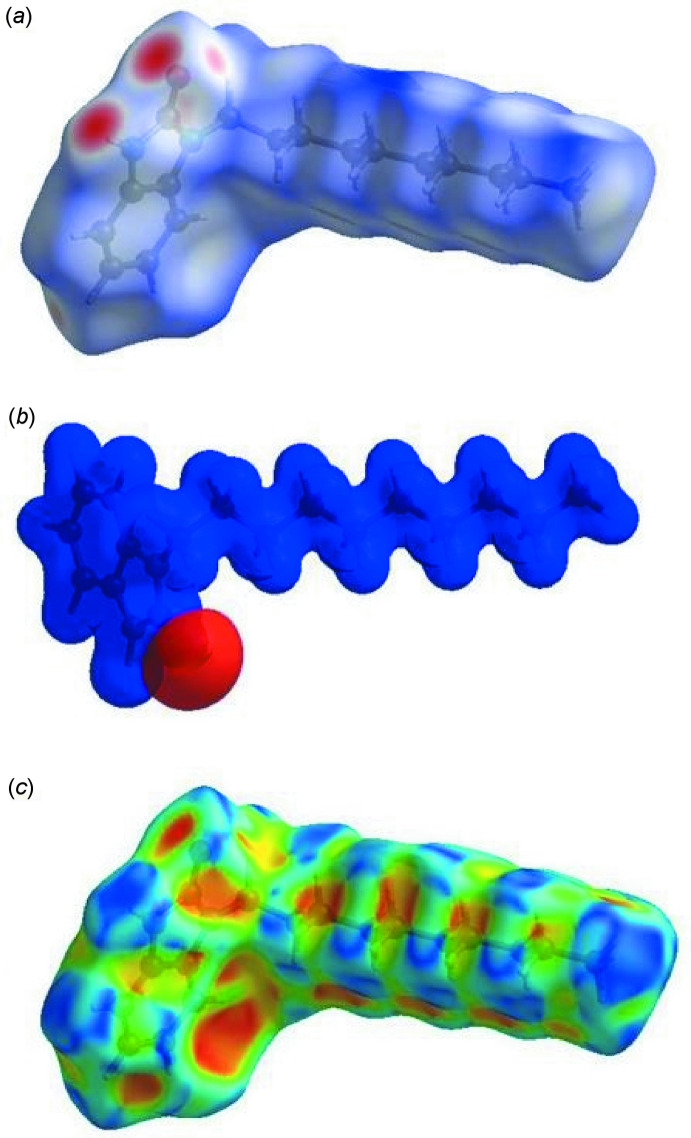
(*a*) View of the three-dimensional Hirshfeld surface of the title compound, plotted over *d*_norm_ in the range of −0.5871 to 1.6590 a.u. (*b*) View of the three-dimensional Hirshfeld surface of the title compound plotted over electrostatic potential energy in the range −0.0500 to 0.0500 a.u. using the STO-3 G basis set at the Hartree–Fock level of theory. (*c*) Hirshfeld surface of the title compound plotted over shape-index.

**Figure 5:**
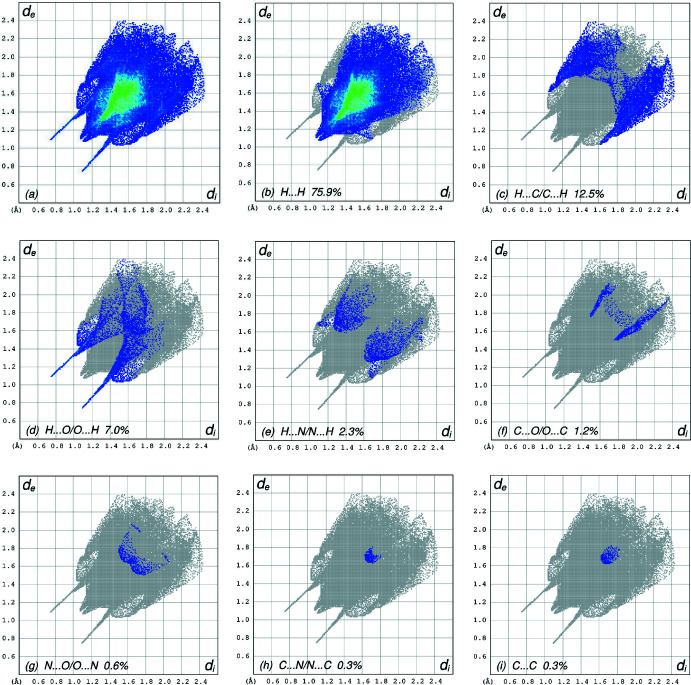
The full two-dimensional fingerprint plots for the title compound, showing (*a*) all inter­actions, and delineated into (*b*) H⋯H, (*c*) H⋯C/C⋯H, (*d*) H⋯O/O⋯H, (*e*) H⋯N/N⋯H, (*f*) C⋯O/O⋯C, (*g*) N⋯O/O⋯N, (*h*) C⋯N/N⋯C and (i) C⋯C inter­actions. The *d*_i_ and *d*_e_ values are the closest inter­nal and external distances (in Å) from given points on the Hirshfeld surface contacts.

In order to visualize the inter­molecular inter­actions in the crystal of the title compound, a Hirshfeld surface (HS) analysis (Hirshfeld, 1977 ▸) was carried out using *Crystal Explorer 17.5* (Turner *et al.*, 2017 ▸). A view of the three-dimensional Hirshfeld surface of (I), plotted over *d*_norm_ and the electrostatic potential map are shown in Fig. 4 ▸ *a* and *b*, respectively. The shape-index of the HS reveals that there are no π–π inter­actions in (I), as shown in Fig. 4 ▸ *c*. The overall two-dimensional fingerprint plot, Fig. 5 ▸ *a*, and those delineated into H⋯H, H⋯C/C⋯H, H⋯O/O⋯H, H⋯N/N⋯H, C⋯O/O⋯C, N⋯O/O⋯N, C⋯N/N⋯C and C⋯C contacts (McKinnon *et al.*, 2007 ▸) are illustrated in Fig. 5 ▸ *b*–*i*, respectively, together with their relative contributions to the Hirshfeld surface. The most important inter­action is H⋯H (Table 2 ▸) contributing 75.9% to the overall crystal packing, which is reflected in Fig. 5 ▸ *b* as widely scattered points of high density due to the large hydrogen content of the mol­ecule, with the tip at *d*_e_ = *d*_i_ = 1.08 Å. In the presence of C—H⋯π inter­actions, the pair of characteristic wings are seen in the fingerprint plot (Fig. 5 ▸ *c*) delineated into H⋯C/C⋯H contacts (12.5% contribution; Table 2 ▸), with the tips at *d*_e_ + *d*_i_ = 2.66 Å. The pair of the scattered points of spikes in the fingerprint plot delineated into H⋯O/O⋯H contacts, Fig. 5 ▸ *d*, with a 7.0% contribution to the HS, has a distribution of points with the tips at *d*_e_ + *d*_i_ = 1.83 Å. The H⋯N/N⋯H contacts, Fig. 5 ▸ *e*, with a 2.3% contribution to the HS have the tips at *d*_e_ + *d*_i_ = 2.92 Å. The C⋯O/O⋯C contacts, Fig. 5 ▸ *f*, with a 1.2% contribution to the HS appear as a pair of scattered points of spikes with the tips at *d*_e_ + *d*_i_ = 3.25 Å. Finally, the N⋯O/O⋯N (Fig. 5 ▸ *g*), N⋯C/C⋯N (Fig. 5 ▸ *h*) and C⋯C (Fig. 5 ▸ *i*) contacts have 0.6%, 0.3% and 0.3% contributions, respectively, to the HS with low-density distributions of points.

**Figure 6:**
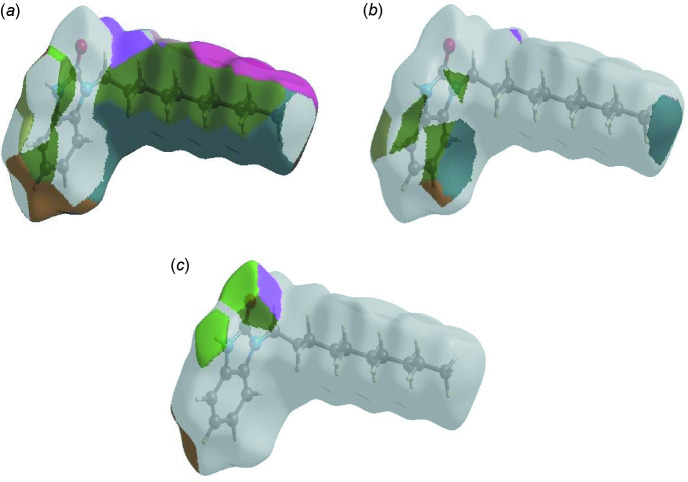
The Hirshfeld surface representations with the function *d*_norm_ plotted onto the surface for (*a*) H⋯H, (*b*) H⋯C/C⋯H and (*c*) H⋯O/O⋯H inter­actions.

The Hirshfeld surface representations with the function *d*_norm_ plotted onto the surface are shown for the H⋯H, H⋯C/C⋯H and H⋯O/O⋯H inter­actions in Fig. 6 ▸ *a*–*c*, respectively.

The Hirshfeld surface analysis confirms the importance of H-atom contacts in establishing the packing. The large number of H⋯H, H⋯C/C⋯H and H⋯O/O⋯H inter­actions suggest that van der Waals inter­actions play the major role in the crystal packing (Hathwar *et al.*, 2015 ▸).

## Inter­action energy calculations   

The inter­molecular inter­action energies were calculated using the CE–B3LYP/6–31G(d,p) energy model available in *Crystal Explorer 17.5* (Turner *et al.*, 2017 ▸), where a cluster of mol­ecules is used by applying crystallographic symmetry operations with respect to a selected central mol­ecule within a default radius of 3.8 Å (Turner *et al.*, 2014 ▸). The total inter­molecular energy (*E*_tot_) is the sum of electrostatic (*E*_ele_), polarization (*E*_pol_), dispersion (*E*_dis_) and exchange-repulsion (*E*_rep_) energies (Turner *et al.*, 2015 ▸) with scale factors of 1.057, 0.740, 0.871 and 0.618, respectively (Mackenzie *et al.*, 2017 ▸). Hydrogen-bonding inter­action energies (in kJ mol^−1^) were calculated as −91.9 (*E*_ele_), −21.4 (*E*_pol_), −14.5 (*E*_dis_), 82.1 (*E*_rep_) and −74.9 (*E*_tot_) for N1—H1⋯O1 and −9.2 (*E*_ele_), −0.6 (*E*_pol_), −65.8 (*E*_dis_), 39.9 (*E*_rep_) and −42.7 (*E*_tot_) for C8—H8*A*⋯O1.

## Database survey   

A search of the Cambridge Structural Database (CSD2021, updated to 2 February, 2021; Groom *et al.*, 2016 ▸) using the fragment below, where *X* = *Y* = H, *R* = (CH_2_)_4_C, found nine similar structures. These are IJUGIE [*X* = *Y* = H, *R* = (CH_2_)_8_CH_3_; Ouzidan *et al.*, 2011*a* ▸], SECBUZ [*X* = *Y* = H, *R* = (CH_2_)_11_CH_3_; Belaziz *et al.*, 2012*b* ▸], ZANXET [*X* = *Y* = H, *R* = (CH_2_)_7_CH_3_; Belaziz *et al.*, 2012*a* ▸], OCAJIN [*X* = H, *Y* = Cl, *R* = (CH_2_)_8_CH_3_; Kandri Rodi *et al.*, 2011 ▸], ULEDEV [*X* = H, *Y* = NO_2_, *R* = (CH_2_)_7_CH_3_; Ouzidan *et al.*, 2011*b* ▸], ULEPIL [*X* = H, *Y* = NO_2_, *R* = (CH_2_)_9_CH_3_; Ouzidan *et al.*, 2011*c* ▸], ULEZAN [*X* = H, *Y* = NO_2_, *R* = (CH_2_)_8_CH_3_; Ouzidan *et al.*, 2011*d* ▸], QUDJAC [*X* = NO_2_, *Y* = H, *R* = (CH_2_)_8_CH_3_; Venkatraman & Fronczek, 2015 ▸] and YAGQII [*X* = NO_2_, *Y* = H, *R* = (CH_2_)_9_CH_3_; Ouzidan *et al.*, 2011*e* ▸]. In all of these mol­ecules, the long alkyl substituent has a straight shape rather than being folded back on itself. This is likely driven by packing considerations as straight alkyl chains can efficiently inter­calate, thereby minimizing void space in the crystal.
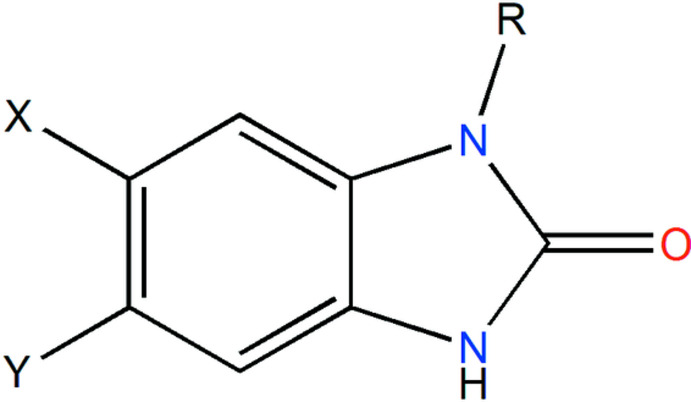


## Synthesis and crystallization   

The title compound was prepared in two steps. In the first step, 1-bromo­decane (11.4 mmol) was added to a mixture of 1-isopropenyl-1*H*-1,3-benzimidazol-2(3*H*)-one (5.7 mmol), potassium hydroxide (5.7 mmol) and tetra-*n*-butyl ammonium bromide (0.15 mmol) in CH_2_Cl_2_ (15 ml). Stirring was continued at room temperature for 48 h. The formed salts were removed by filtration, and the filtrate was concentrated under reduced pressure. The residue obtained was purified by recrystallization from ethanol to obtain 1-(prop-1-en-2-yl)-3-decyl-2,3di­hydro-1*H*-benzimidazol-2(3*H*)-one in 82% yield. In the second step, 1-(prop-1-en-2-yl)-3-decyl-2,3-di­hydro-1*H*-benzimidazol-2-one (7.0 mmol) was dissolved in a mixture of di­methyl­formamide (DMF; 10 ml) and cold sulfuric acid (15 ml, 50%_wt_). The reaction mixture was stirred at room temperature for 12 h. The precipitate obtained was filtered off and washed with water and subsequently dried. The resulting residue was purified by recrystallization from ethanol to obtain colourless crystals in 75% yield.

^1^H NMR (300 MHz, DMSO-*d*_6_): 0.87 (*t*, 3H, CH_3_); 1.25–1.67 (*m*, 16H, CH_2_); 2.80–3.04 (*m*, 2H, CH_2_); 6.99–7.12 (*m*, 4H, H_arom_); 10.58 (*s*,1H, NH). ^13^C NMR (75 MHz, DMSO-*d*_6_): 14.14 (CH_3_); 22.70, 26.90, 28.44, 29.31, 29.51, 29.56, 29.74, 31.90, 41.44 (CH_2_); 107.84, 108.45, 121.20, 121.65 (CH_arom_); 128.52, 129.64 (Cq), 153.43 (C=O).

## Refinement   

Crystal, data collection and refinement details are presented in Table 3 ▸. Hydrogen atoms were located in difference-Fourier maps and were freely refined.

## Supplementary Material

Crystal structure: contains datablock(s) I, global. DOI: 10.1107/S2056989021004291/wm5606sup1.cif

Structure factors: contains datablock(s) I. DOI: 10.1107/S2056989021004291/wm5606Isup2.hkl

Supporting information file. DOI: 10.1107/S2056989021004291/wm5606Isup3.cdx

Supporting information file. DOI: 10.1107/S2056989021004291/wm5606Isup4.cml

CCDC reference: 2079158

Additional supporting information:  crystallographic information; 3D view; checkCIF report

## Figures and Tables

**Table 1:** Hydrogen-bond geometry (Å, °) *Cg*2 is the centroid of the C1–C6 ring.

*D*—H⋯*A*	*D*—H	H⋯*A*	*D*⋯*A*	*D*—H⋯*A*
N1—H1⋯O1^ii^	0.923 (16)	1.932 (16)	2.8393 (12)	167.0 (13)
C8—H8*A*⋯O1^viii^	0.995 (13)	2.573 (13)	3.4648 (12)	149.1 (9)
C17—H17*C*⋯*Cg*2^v^	1.00 (2)	2.985 (19)	3.6656 (17)	126.0 (14)

Symmetry codes: (ii) -x, -y+1, -z+1; (v) -x+1, -y+1, -z+1; (viii) x, y-1, z.

**Table 2:** Selected interatomic distances (Å)

O1⋯C1^i^	3.2784 (12)	H9*B*⋯H11*B*	2.53 (2)
O1⋯N1^ii^	2.8394 (11)	H10*A*⋯H12*A*	2.55 (2)
C4⋯O1^iii^	3.2820 (14)	H10*B*⋯H12*B*	2.58 (2)
O1⋯H8*B*	2.486 (11)	H11*A*⋯H13*A*	2.58 (2)
O1⋯H1^ii^	1.934 (16)	H11*A*⋯H16*A* ^v^	2.43 (2)
O1⋯H8*A* ^iv^	2.571 (11)	H11*B*⋯H13*B*	2.51 (2)
H4⋯O1^iii^	2.417 (13)	H12*A*⋯H14*A*	2.55 (2)
N1⋯H8*A* ^iv^	2.878 (12)	H12*A*⋯H15*A* ^v^	2.57 (2)
N1⋯H8*B* ^i^	2.949 (12)	H12*B*⋯H14*B*	2.53 (2)
N2⋯H10*A*	2.843 (14)	H13*A*⋯H15*A*	2.55 (2)
C7⋯C7^i^	3.2937 (14)	H13*A*⋯H14*A* ^v^	2.52 (2)
C2⋯H17*C* ^v^	2.90 (2)	H13*B*⋯H15*B*	2.57 (2)
C7⋯H1^ii^	2.828 (16)	H13*B*⋯H16*B* ^vii^	2.47 (2)
C7⋯H8*A* ^iv^	2.774 (11)	H14*A*⋯H16*A*	2.51 (2)
H2⋯H9*A*	2.572 (19)	H14*B*⋯H16*B*	2.56 (2)
H2⋯H17*A* ^vi^	2.34 (2)	H14*B*⋯H16*B* ^vii^	2.54 (2)
H8*B*⋯H10*B*	2.507 (18)	H15*A*⋯H17*A*	2.60 (2)
H9*A*⋯H11*A*	2.550 (19)	H15*B*⋯H17*B*	2.54 (2)

Symmetry codes: (i) -x, -y, -z+1; (ii) -x, -y+1, -z+1; (iii) x, -y+{\script{1\over 2}}, z+{\script{1\over 2}}; (iv) x, y+1, z; (v) -x+1, -y+1, -z+1; (vi) -x+1, -y, -z+1; (vii) -x+1, y-{\script{1\over 2}}, -z+{\script{1\over 2}}.

**Table 3:** Experimental details

Crystal data	
Chemical formula	C_17_H_26_N_2_O
*M*_r_	274.40
Crystal system, space group	Monoclinic, *P*2_1_/*c*
Temperature (K)	150
*a*, *b*, *c* (Å)	17.3256 (5), 5.5662 (2), 16.7244 (5)
β (°)	97.433 (1)
*V* (Å^3^)	1599.31 (9)
*Z*	4
Radiation type	Cu *K*α
μ (mm^−1^)	0.55
Crystal size (mm)	0.26 × 0.17 × 0.10

Data collection	
Diffractometer	Bruker D8 VENTURE PHOTON 100 CMOS
Absorption correction	Numerical (*SADABS*; Krause *et al.*, 2015 ▸)
*T*_min_, *T*_max_	0.88, 0.95
No. of measured, independent and observed [*I* > 2σ(*I*)] reflections	11457, 3082, 2857
*R*_int_	0.028
(sin θ/λ)_max_ (Å^−1^)	0.618

Refinement	
*R*[*F* ^2^ > 2σ(*F* ^2^)], *wR*(*F* ^2^), *S*	0.041, 0.101, 1.08
No. of reflections	3082
No. of parameters	286
H-atom treatment	All H-atom parameters refined
Δρ_max_, Δρ_min_ (e Å^−3^)	0.19, −0.26

Computer programs: *APEX3* and *SAINT* (Bruker, 2016 ▸), *SAINT* (Bruker, 2016 ▸), *SHELXT/5* (Sheldrick, 2015*a* ▸), *SHELXL2018/3* (Sheldrick, 2015*b* ▸), *DIAMOND* (Brandenburg & Putz, 2012 ▸) and *publCIF* (Westrip, 2010 ▸).

## supplementary crystallographic information

### Crystal data

**Table:**

C_17_H_26_N_2_O	*F*(000) = 600
*M**_r_* = 274.40	*D*_x_ = 1.140 Mg m^−^^3^
Monoclinic, *P*2_1_/*c*	Cu *K*α radiation, λ = 1.54178 Å
*a* = 17.3256 (5) Å	Cell parameters from 9927 reflections
*b* = 5.5662 (2) Å	θ = 2.6–72.4°
*c* = 16.7244 (5) Å	µ = 0.55 mm^−^^1^
β = 97.433 (1)°	*T* = 150 K
*V* = 1599.31 (9) Å^3^	Parallelepiped, colourless
*Z* = 4	0.26 × 0.17 × 0.10 mm

### Data collection

**Table:**

Bruker D8 VENTURE PHOTON 100 CMOS diffractometer	3082 independent reflections
Radiation source: INCOATEC IµS micro–focus source	2857 reflections with *I* > 2σ(*I*)
Mirror monochromator	*R*_int_ = 0.028
Detector resolution: 10.4167 pixels mm^-1^	θ_max_ = 72.3°, θ_min_ = 5.6°
ω scans	*h* = −19→21
Absorption correction: numerical (*SADABS*; Krause *et al.*, 2015)	*k* = −6→6
*T*_min_ = 0.88, *T*_max_ = 0.95	*l* = −20→17
11457 measured reflections	

### Refinement

**Table:**

Refinement on *F*^2^	Secondary atom site location: difference Fourier map
Least-squares matrix: full	Hydrogen site location: difference Fourier map
*R*[*F*^2^ > 2σ(*F*^2^)] = 0.041	All H-atom parameters refined
*wR*(*F*^2^) = 0.101	*w* = 1/[σ^2^(*F*_o_^2^) + (0.0505*P*)^2^ + 0.3296*P*] where *P* = (*F*_o_^2^ + 2*F*_c_^2^)/3
*S* = 1.08	(Δ/σ)_max_ = 0.001
3082 reflections	Δρ_max_ = 0.19 e Å^−^^3^
286 parameters	Δρ_min_ = −0.26 e Å^−^^3^
0 restraints	Extinction correction: *SHELXL 2018/3* (Sheldrick, 2015*b*), Fc^*^=kFc[1+0.001xFc^2^λ^3^/sin(2θ)]^-1/4^
Primary atom site location: dual	Extinction coefficient: 0.0415 (17)

### Special details

**Table:**

Geometry. All esds (except the esd in the dihedral angle between two l.s. planes) are estimated using the full covariance matrix. The cell esds are taken into account individually in the estimation of esds in distances, angles and torsion angles; correlations between esds in cell parameters are only used when they are defined by crystal symmetry. An approximate (isotropic) treatment of cell esds is used for estimating esds involving l.s. planes.
Refinement. Refinement of F^2^ against ALL reflections. The weighted R-factor wR and goodness of fit S are based on F^2^, conventional R-factors R are based on F, with F set to zero for negative F^2^. The threshold expression of F^2^ > 2sigma(F^2^) is used only for calculating R-factors(gt) etc. and is not relevant to the choice of reflections for refinement. R-factors based on F^2^ are statistically about twice as large as those based on F, and R- factors based on ALL data will be even larger.

### Fractional atomic coordinates and isotropic or equivalent isotropic displacement parameters (Å^2^)

**Table:**

	*x*	*y*	*z*	*U*_iso_*/*U*_eq_	
O1	0.04776 (4)	0.28130 (13)	0.44474 (4)	0.0303 (2)	
N1	0.05137 (5)	0.31336 (16)	0.58449 (5)	0.0277 (2)	
H1	0.0208 (9)	0.449 (3)	0.5834 (8)	0.049 (4)*	
N2	0.11079 (5)	0.00636 (15)	0.53573 (5)	0.0257 (2)	
C1	0.11866 (6)	−0.02174 (17)	0.61924 (6)	0.0255 (2)	
C2	0.15374 (6)	−0.20007 (19)	0.66878 (7)	0.0303 (3)	
H2	0.1796 (8)	−0.337 (3)	0.6474 (8)	0.039 (3)*	
C3	0.15165 (7)	−0.1745 (2)	0.75149 (7)	0.0337 (3)	
H3	0.1783 (8)	−0.300 (2)	0.7883 (8)	0.042 (4)*	
C4	0.11570 (7)	0.0218 (2)	0.78254 (7)	0.0332 (3)	
H4	0.1157 (8)	0.039 (2)	0.8423 (8)	0.037 (3)*	
C5	0.07947 (6)	0.19953 (19)	0.73228 (6)	0.0302 (3)	
H5	0.0528 (7)	0.334 (2)	0.7533 (7)	0.033 (3)*	
C6	0.08152 (6)	0.17382 (17)	0.65026 (6)	0.0261 (2)	
C7	0.06765 (6)	0.20902 (17)	0.51435 (6)	0.0253 (2)	
C8	0.13586 (6)	−0.16239 (18)	0.47772 (6)	0.0284 (3)	
H8A	0.1186 (7)	−0.327 (2)	0.4909 (7)	0.031 (3)*	
H8B	0.1074 (7)	−0.116 (2)	0.4234 (8)	0.030 (3)*	
C9	0.22347 (6)	−0.16205 (19)	0.47500 (7)	0.0308 (3)	
H9A	0.2512 (8)	−0.205 (2)	0.5293 (8)	0.035 (3)*	
H9B	0.2344 (8)	−0.290 (2)	0.4382 (8)	0.035 (3)*	
C10	0.25501 (6)	0.0740 (2)	0.44707 (7)	0.0325 (3)	
H10A	0.2498 (8)	0.199 (3)	0.4881 (8)	0.042 (4)*	
H10B	0.2229 (8)	0.128 (2)	0.3965 (8)	0.038 (3)*	
C11	0.33982 (7)	0.0550 (2)	0.43186 (7)	0.0355 (3)	
H11A	0.3717 (8)	−0.020 (2)	0.4812 (8)	0.041 (3)*	
H11B	0.3434 (8)	−0.060 (3)	0.3859 (8)	0.042 (4)*	
C12	0.37542 (7)	0.2942 (2)	0.41212 (8)	0.0371 (3)	
H12A	0.3739 (8)	0.409 (3)	0.4593 (8)	0.044 (4)*	
H12B	0.3426 (8)	0.369 (3)	0.3659 (8)	0.040 (3)*	
C13	0.45872 (7)	0.2725 (2)	0.39272 (8)	0.0374 (3)	
H13A	0.4913 (9)	0.197 (3)	0.4389 (9)	0.045 (4)*	
H13B	0.4594 (8)	0.161 (3)	0.3467 (9)	0.044 (4)*	
C14	0.49438 (7)	0.5123 (2)	0.37418 (8)	0.0383 (3)	
H14A	0.4944 (9)	0.622 (3)	0.4218 (9)	0.050 (4)*	
H14B	0.4604 (8)	0.589 (3)	0.3278 (8)	0.042 (4)*	
C15	0.57697 (7)	0.4932 (2)	0.35263 (8)	0.0383 (3)	
H15A	0.6106 (9)	0.409 (3)	0.3983 (9)	0.047 (4)*	
H15B	0.5772 (8)	0.385 (3)	0.3040 (8)	0.044 (4)*	
C16	0.61285 (7)	0.7340 (2)	0.33601 (8)	0.0416 (3)	
H16A	0.6112 (9)	0.842 (3)	0.3852 (9)	0.054 (4)*	
H16B	0.5799 (10)	0.814 (3)	0.2910 (9)	0.053 (4)*	
C17	0.69572 (9)	0.7119 (3)	0.31580 (10)	0.0540 (4)	
H17A	0.7312 (11)	0.646 (3)	0.3640 (11)	0.072 (5)*	
H17B	0.6977 (10)	0.598 (3)	0.2673 (10)	0.066 (5)*	
H17C	0.7161 (11)	0.872 (4)	0.3005 (11)	0.076 (5)*	

### Atomic displacement parameters (Å^2^)

**Table:**

	*U*^11^	*U*^22^	*U*^33^	*U*^12^	*U*^13^	*U*^23^
O1	0.0355 (4)	0.0285 (4)	0.0273 (4)	0.0019 (3)	0.0055 (3)	0.0041 (3)
N1	0.0307 (4)	0.0240 (4)	0.0289 (5)	0.0026 (3)	0.0066 (3)	0.0010 (3)
N2	0.0292 (4)	0.0237 (4)	0.0251 (4)	0.0005 (3)	0.0063 (3)	0.0006 (3)
C1	0.0261 (5)	0.0245 (5)	0.0264 (5)	−0.0041 (4)	0.0051 (4)	−0.0002 (4)
C2	0.0343 (5)	0.0253 (5)	0.0315 (6)	0.0005 (4)	0.0049 (4)	0.0011 (4)
C3	0.0391 (6)	0.0307 (6)	0.0307 (6)	−0.0021 (4)	0.0022 (4)	0.0053 (4)
C4	0.0376 (6)	0.0359 (6)	0.0267 (6)	−0.0055 (4)	0.0061 (4)	0.0006 (4)
C5	0.0329 (5)	0.0288 (5)	0.0301 (6)	−0.0031 (4)	0.0087 (4)	−0.0032 (4)
C6	0.0255 (5)	0.0246 (5)	0.0284 (5)	−0.0035 (4)	0.0049 (4)	0.0012 (4)
C7	0.0255 (5)	0.0231 (5)	0.0279 (5)	−0.0033 (4)	0.0061 (4)	0.0011 (4)
C8	0.0337 (5)	0.0235 (5)	0.0288 (5)	−0.0009 (4)	0.0071 (4)	−0.0030 (4)
C9	0.0340 (6)	0.0294 (6)	0.0299 (6)	0.0050 (4)	0.0072 (4)	0.0000 (4)
C10	0.0321 (5)	0.0309 (6)	0.0355 (6)	0.0020 (4)	0.0080 (5)	0.0003 (4)
C11	0.0336 (6)	0.0365 (6)	0.0376 (6)	0.0025 (5)	0.0090 (5)	0.0021 (5)
C12	0.0339 (6)	0.0375 (6)	0.0408 (7)	−0.0002 (5)	0.0081 (5)	0.0009 (5)
C13	0.0347 (6)	0.0385 (6)	0.0399 (7)	0.0005 (5)	0.0086 (5)	0.0020 (5)
C14	0.0355 (6)	0.0377 (7)	0.0423 (7)	−0.0010 (5)	0.0070 (5)	−0.0013 (5)
C15	0.0366 (6)	0.0370 (6)	0.0423 (7)	−0.0022 (5)	0.0081 (5)	−0.0007 (5)
C16	0.0389 (6)	0.0401 (7)	0.0454 (7)	−0.0055 (5)	0.0037 (5)	−0.0002 (5)
C17	0.0432 (7)	0.0588 (9)	0.0612 (9)	−0.0130 (6)	0.0111 (7)	0.0003 (7)

### Geometric parameters (Å, º)

**Table:**

O1—C7	1.2378 (12)	C10—H10A	0.988 (14)
N1—C7	1.3706 (13)	C10—H10B	0.996 (14)
N1—C6	1.3920 (13)	C11—C12	1.5214 (16)
N1—H1	0.923 (16)	C11—H11A	1.021 (14)
N2—C7	1.3747 (13)	C11—H11B	1.009 (14)
N2—C1	1.3944 (13)	C12—C13	1.5245 (16)
N2—C8	1.4564 (12)	C12—H12A	1.017 (14)
C1—C2	1.3823 (14)	C12—H12B	0.989 (14)
C1—C6	1.3981 (14)	C13—C14	1.5197 (16)
C2—C3	1.3958 (16)	C13—H13A	0.990 (15)
C2—H2	0.974 (14)	C13—H13B	0.990 (15)
C3—C4	1.3910 (16)	C14—C15	1.5239 (16)
C3—H3	1.003 (14)	C14—H14A	1.004 (16)
C4—C5	1.3940 (16)	C14—H14B	1.007 (14)
C4—H4	1.004 (13)	C15—C16	1.5182 (17)
C5—C6	1.3841 (15)	C15—H15A	1.014 (15)
C5—H5	0.970 (13)	C15—H15B	1.013 (15)
C8—C9	1.5246 (15)	C16—C17	1.5221 (19)
C8—H8A	0.995 (13)	C16—H16A	1.020 (16)
C8—H8B	1.009 (13)	C16—H16B	0.989 (16)
C9—C10	1.5195 (15)	C17—H17A	1.017 (19)
C9—H9A	1.000 (13)	C17—H17B	1.033 (18)
C9—H9B	0.974 (13)	C17—H17C	1.00 (2)
C10—C11	1.5269 (15)		
			
O1···N2^i^	3.2324 (11)	H8B···H10B	2.507 (18)
O1···C1^i^	3.2784 (12)	H9A···H11A	2.550 (19)
O1···N1^ii^	2.8394 (11)	H9B···H11B	2.53 (2)
C4···O1^iii^	3.2820 (14)	H10A···H12A	2.55 (2)
O1···H8B	2.486 (11)	H10B···H12B	2.58 (2)
O1···H1^ii^	1.934 (16)	H11A···H13A	2.58 (2)
O1···H8A^iv^	2.571 (11)	H11A···H16A^v^	2.43 (2)
H4···O1^iii^	2.417 (13)	H11B···H13B	2.51 (2)
N1···C2^iv^	3.4382 (14)	H12A···H14A	2.55 (2)
N1···C8^i^	3.3820 (14)	H12A···H15A^v^	2.57 (2)
N2···C7^i^	3.3206 (14)	H12B···H14B	2.53 (2)
N1···H8A^iv^	2.878 (12)	H13A···H15A	2.55 (2)
N1···H8B^i^	2.949 (12)	H13A···H14A^v^	2.52 (2)
N2···H10A	2.843 (14)	H13B···H15B	2.57 (2)
C7···C8^i^	3.5550 (15)	H13B···H16B^vii^	2.47 (2)
C7···C7^i^	3.2937 (14)	H14A···H16A	2.51 (2)
C2···H17C^v^	2.90 (2)	H14B···H16B	2.56 (2)
C7···H1^ii^	2.828 (16)	H14B···H16B^vii^	2.54 (2)
C7···H8A^iv^	2.774 (11)	H15A···H17A	2.60 (2)
H2···H9A	2.572 (19)	H15B···H17B	2.54 (2)
H2···H17A^vi^	2.34 (2)		
			
C7—N1—C6	110.01 (9)	H10A—C10—H10B	106.7 (11)
C7—N1—H1	120.8 (9)	C12—C11—C10	113.68 (10)
C6—N1—H1	129.0 (9)	C12—C11—H11A	109.9 (8)
C7—N2—C1	109.39 (8)	C10—C11—H11A	109.0 (8)
C7—N2—C8	123.71 (8)	C12—C11—H11B	109.0 (8)
C1—N2—C8	126.57 (8)	C10—C11—H11B	109.0 (8)
C2—C1—N2	131.25 (9)	H11A—C11—H11B	106.0 (11)
C2—C1—C6	121.63 (9)	C11—C12—C13	113.47 (10)
N2—C1—C6	107.11 (8)	C11—C12—H12A	109.2 (8)
C1—C2—C3	117.07 (10)	C13—C12—H12A	109.6 (8)
C1—C2—H2	121.9 (8)	C11—C12—H12B	109.1 (8)
C3—C2—H2	121.0 (8)	C13—C12—H12B	109.3 (8)
C4—C3—C2	121.36 (10)	H12A—C12—H12B	105.9 (11)
C4—C3—H3	120.8 (8)	C14—C13—C12	113.26 (10)
C2—C3—H3	117.9 (8)	C14—C13—H13A	109.1 (8)
C3—C4—C5	121.38 (10)	C12—C13—H13A	109.1 (8)
C3—C4—H4	120.4 (7)	C14—C13—H13B	110.2 (8)
C5—C4—H4	118.3 (7)	C12—C13—H13B	108.7 (8)
C6—C5—C4	117.19 (10)	H13A—C13—H13B	106.1 (12)
C6—C5—H5	120.9 (7)	C13—C14—C15	113.93 (10)
C4—C5—H5	121.9 (7)	C13—C14—H14A	109.3 (9)
C5—C6—N1	132.10 (10)	C15—C14—H14A	109.1 (9)
C5—C6—C1	121.36 (9)	C13—C14—H14B	108.6 (8)
N1—C6—C1	106.55 (9)	C15—C14—H14B	108.7 (8)
O1—C7—N1	127.16 (10)	H14A—C14—H14B	106.9 (12)
O1—C7—N2	125.96 (9)	C16—C15—C14	113.62 (10)
N1—C7—N2	106.88 (8)	C16—C15—H15A	109.7 (9)
N2—C8—C9	113.76 (9)	C14—C15—H15A	108.7 (8)
N2—C8—H8A	108.7 (7)	C16—C15—H15B	109.5 (8)
C9—C8—H8A	109.7 (7)	C14—C15—H15B	109.4 (8)
N2—C8—H8B	106.3 (7)	H15A—C15—H15B	105.7 (12)
C9—C8—H8B	110.2 (7)	C15—C16—C17	112.95 (11)
H8A—C8—H8B	108.0 (10)	C15—C16—H16A	108.4 (9)
C10—C9—C8	114.16 (9)	C17—C16—H16A	110.6 (9)
C10—C9—H9A	109.6 (7)	C15—C16—H16B	109.0 (9)
C8—C9—H9A	109.6 (8)	C17—C16—H16B	109.6 (9)
C10—C9—H9B	109.2 (8)	H16A—C16—H16B	106.0 (13)
C8—C9—H9B	107.1 (8)	C16—C17—H17A	110.1 (11)
H9A—C9—H9B	106.9 (11)	C16—C17—H17B	110.6 (10)
C9—C10—C11	112.56 (9)	H17A—C17—H17B	108.8 (14)
C9—C10—H10A	108.9 (8)	C16—C17—H17C	111.0 (11)
C11—C10—H10A	109.9 (8)	H17A—C17—H17C	109.1 (15)
C9—C10—H10B	109.7 (8)	H17B—C17—H17C	107.1 (14)
C11—C10—H10B	108.8 (8)		
			
C7—N2—C1—C2	177.13 (10)	C6—N1—C7—O1	177.92 (9)
C8—N2—C1—C2	3.56 (17)	C6—N1—C7—N2	−2.09 (11)
C7—N2—C1—C6	−2.02 (11)	C1—N2—C7—O1	−177.48 (9)
C8—N2—C1—C6	−175.60 (9)	C8—N2—C7—O1	−3.68 (15)
N2—C1—C2—C3	179.89 (10)	C1—N2—C7—N1	2.53 (11)
C6—C1—C2—C3	−1.06 (15)	C8—N2—C7—N1	176.33 (8)
C1—C2—C3—C4	0.11 (16)	C7—N2—C8—C9	111.38 (11)
C2—C3—C4—C5	0.78 (17)	C1—N2—C8—C9	−75.91 (12)
C3—C4—C5—C6	−0.69 (16)	N2—C8—C9—C10	−63.87 (12)
C4—C5—C6—N1	179.37 (10)	C8—C9—C10—C11	−170.56 (9)
C4—C5—C6—C1	−0.26 (15)	C9—C10—C11—C12	−174.11 (10)
C7—N1—C6—C5	−178.81 (10)	C10—C11—C12—C13	−177.01 (10)
C7—N1—C6—C1	0.86 (11)	C11—C12—C13—C14	−179.20 (10)
C2—C1—C6—C5	1.17 (15)	C12—C13—C14—C15	−178.58 (10)
N2—C1—C6—C5	−179.58 (9)	C13—C14—C15—C16	−178.72 (11)
C2—C1—C6—N1	−178.55 (9)	C14—C15—C16—C17	179.15 (11)
N2—C1—C6—N1	0.70 (10)		

Symmetry codes: (i) −*x*, −*y*, −*z*+1; (ii) −*x*, −*y*+1, −*z*+1; (iii) *x*, −*y*+1/2, *z*+1/2; (iv) *x*, *y*+1, *z*; (v) −*x*+1, −*y*+1, −*z*+1; (vi) −*x*+1, −*y*, −*z*+1; (vii) −*x*+1, *y*−1/2, −*z*+1/2.

### Hydrogen-bond geometry (Å, º)

*Cg*2 is the centroid of the C1–C6 ring.

**Table:**

*D*—H···*A*	*D*—H	H···*A*	*D*···*A*	*D*—H···*A*
N1—H1···O1^ii^	0.923 (16)	1.932 (16)	2.8393 (12)	167.0 (13)
C8—H8*A*···O1^viii^	0.995 (13)	2.573 (13)	3.4648 (12)	149.1 (9)
C17—H17*C*···*Cg*2^v^	1.00 (2)	2.985 (19)	3.6656 (17)	126.0 (14)

Symmetry codes: (ii) −*x*, −*y*+1, −*z*+1; (v) −*x*+1, −*y*+1, −*z*+1; (viii) *x*, *y*−1, *z*.
